# The impact of the COVID-19 pandemic on the provision & utilisation of primary health care services in Goma, Democratic Republic of the Congo, Kambia district, Sierra Leone & Masaka district, Uganda

**DOI:** 10.1371/journal.pone.0286295

**Published:** 2023-06-02

**Authors:** K. Kasonia, D. Tindanbil, J. Kitonsa, K. Baisley, F. Zalwango, L. Enria, A. Mansaray, M. James, Y. Nije, D. Tetsa Tata, B. J. Lawal, A. Drammeh, B. Lowe, D. Mukadi-Bamuleka, S. Mounier-Jack, F. Nakiyimba, P. Obady, J. Muhavi, J. S. Bangura, B. Greenwood, M. Samai, B. Leigh, D. Watson-Jones, H. Kavunga-Membo, E. Ruzagira, K. E. Gallagher

**Affiliations:** 1 LSHTM-INRB Research Partnership, Goma, Democratic Republic of the Congo; 2 LSHTM-COMAHS Research Partnership, Kambia, Sierra Leone; 3 MRC/UVRI and LSHTM Uganda Research Unit, Entebbe, Uganda; 4 London School of Hygiene & Tropical Medicine (LSHTM), London, United Kingdom; 5 Laboratoire Rodolphe-Merieux, Institut National de Recherche Biomédicale (INRB-Goma), Goma, Democratic Republic of the Congo; 6 Ministry of Health, Masaka, Masaka District, Uganda; 7 Ministry of Health, Goma, Democratic Republic of Congo; 8 University of Sierra Leone College of Medicine and Allied Health Sciences (COMAHS), Freetown, Sierra Leone; 9 Ministry of Health, Kambia, Kambia District, Sierra Leone; 10 Mwanza Intervention Trials Unit, National Institute for Medical Research, Mwanza, Tanzania; 11 KEMRI-Wellcome Trust Research Programme, Kilifi, Kenya; University of Ghana Business School, GHANA

## Abstract

**Introduction:**

This study aimed to determine whether the COVID-19 pandemic had an impact on essential primary healthcare services at public primary healthcare facilities.

**Methods:**

The number of weekly consultations for antenatal care (ANC), outpatient (OPD), immunisations (EPI), family planning (FP) and HIV services, between January 2018 and December 2020, were collected from 25 facilities in Masaka district, Uganda, 21 in Goma, and 29 in Kambia district, Sierra Leone. Negative binomial regression models accounting for clustering and season were used to analyse changes in activity levels between 2018, 2019 and 2020.

**Results:**

In Goma, we found no change in OPD, EPI or ANC consultations, FP was 17% lower in March-July 2020 compared to 2019, but this recovered by December 2020. New diagnoses of HIV were 34% lower throughout 2020 compared to 2019. In Sierra Leone, compared to the same periods in 2019, facilities had 18–29% fewer OPD consultations throughout 2020, and 27% fewer DTP3 doses in March-July 2020. There was no evidence of differences in other services. In Uganda there were 20–35% fewer under-5 OPD consultations, 21–66% fewer MCV1 doses, and 48–51% fewer new diagnoses of HIV throughout 2020, compared to 2019. There was no difference in the number of HPV doses delivered.

**Conclusions:**

The level of disruption varied across the different settings and qualitatively appeared to correlate with the strength of lockdown measures and reported attitudes towards the risk posed by COVID-19. Mitigation strategies such as health communications campaigns and outreach services may be important to limit the impact of lockdowns on primary healthcare services.

## Introduction

The World Health Organization (WHO) pulse surveys reported that the COVID-19 pandemic affected the provision and utilization of essential primary healthcare services in >90% of countries worldwide. In July 2020, nearly all countries reported either partial (5%-50%) or severe (>50%) change in service provision or use. Low and lower-middle income countries were more affected than countries in higher income brackets [1]. In March 2021, 94% of the 135 countries that took part in the survey reported residual service disruption [2]. In response to the pandemic, various containment measures such as social distancing, lockdowns, curfews, closure of schools and bans on gatherings were instituted across the globe [3]. Fewer transport options and less disposable income during lockdowns, alongside fears and misconceptions around the risk and feasibility of accessing services could have reduced utilisation of primary care. Healthcare resources, including staff, facilities, consumables, treatments, personal protective equipment, were re-prioritised to fight the pandemic, social distancing measures were put in place in facilities, and many of the workforce fell sick, reducing the capacity to provide essential health services in many settings [1].

Literature reviews have already documented that COVID-19 had a considerable impact on primary healthcare at both the service and patient level; however, almost all studies are from Europe or the USA [4–6]. There is a paucity of information about the impact of the pandemic on the provision and utilization of primary healthcare services in low- and middle-income countries (LMICs), especially in Africa. A study in Rwanda documented a significant decrease in utilization of antenatal care, facility-based deliveries, post-natal care and vaccinations when comparing April-May 2020 with April-May 2019 [7]. In Uganda, a 75% reduction in HIV testing and initiation of antiretroviral treatment was reported in the first three weeks of April 2020 compared to the weekly average for the period from Jan-Mar 2020 [8].

The first case of SARS-CoV-2 infection in the Democratic Republic of the Congo (DRC) was identified on 10^th^ March 2020. From the 18^th^ March onwards, in response to the pandemic, the government put in place movement restrictions, closure of public spaces, limitations on gatherings and compulsory wearing of masks in public [9]. The first case of SARS-CoV-2 infection in Sierra Leone was recorded on 30^th^ March 2020 [10], the government imposed a dusk to dawn curfew, movement restrictions, bans on public gatherings, school closures, and compulsory wearing of masks. The first case of SARS-CoV-2 infection was confirmed in Uganda on 21^st^ March 2020 [11]. Subsequently, the Ministry of Health imposed movement restrictions, closure of public spaces and schools, gatherings were limited, and masks were made compulsory in public. All these measures may have affected effective provision and utilization of primary health services in these countries.

We aimed to determine to what extent the pandemic impacted provision and utilization of primary healthcare services in 2020 in three distinct settings with different documented burdens of COVID-19 and different lockdown measures. A French translation of this manuscript is provided in S2 File.

## Methods

### Study setting & health facility selection

The study was conducted in three areas, Goma, in the Democratic Republic of the Congo, Kambia District, north-western Sierra Leone, and Masaka District, south-western Uganda (Tables 1 and 2) by research partners with a record of prior collaboration on Ebola Vaccine Projects. District/ regional authorities were approached for approval to conduct the study and complete lists of health facilities in each area were compiled. Private health facilities were excluded from selection, as the aim of the project was to inform the provision of public health services. A selection of health centres was made to include all 25 available government health centres in Masaka, all 21 accessible health centres in urban Goma; and a random number generator was used to select a representative selection of 29 health facilities in Kambia, proportional to the total number of health posts and health centres in the district.

**Table 1:** The study setting and selected health centres.

Country	Selected region/ district	Description and total population	Total public health facilities in the area	Selected facilities for quantitative data collection	Selected facilities for qualitative interviews
**DRC**	Goma	Urban and suburban; estimated population: 600,000–1 million	39 (26 health centres, 13 tertiary care hospitals)	21 (21 health centres^1^)	12
**Sierrra Leone**	Kambia	Suburban and rural; estimated population: 350,000	68 (55 health posts, 15 health centres, 1 hospital)	29 (22 health posts, 6 health centres, 1 hospital with a primary health care dept.^2^)	15
**Uganda**	Masaka	Rural; estimated population: 307,000	26 (25 health facilities, 1 referral Hospital)	25 (14 level II, 9 level III, 2 level IV health centres^3^)	15

^1^ In Goma, health centres provide primary healthcare to the urban population; 21 of the 26 public health centres were selected due to security and logistical constraints during data collection.

^2^ In Kambia, health posts, community health centres and some hospitals provide primary care to the population. Health posts (community or maternal child health posts) are small and provide services in remote rural settings; community health centres are larger, based in central locations within each chiefdom and can have laboratory and more consistent cold chain services. Kambia district hospital was included as the dominant public primary health care provider within Kambia town.

^3^ In Masaka, level II health facilities provide outpatient services to the surrounding parish, Level III facilities are slightly larger sub-county facilities which may have maternity wards, and level IV facilities have inpatient and outpatient services.

**Table 2:** Definition of periods of analysis based on the COVID pandemic and lockdown measures.

Area	Period	Approx. dates in the period (2018–20) ^1^	N (weeks/ year)	Description of lockdown measures in place in 2020	Mean lockdown stringency index for the period [3]	National number of reported COVID-19 cases [14]	National number of cases per 1 million population
Goma	0	1^st^ January—22^nd^ March	12	Pre-COVID	0		
	1	23rd March—19^th^ July	17	First wave of reported cases peaks. State of emergency (declared 18^th^ March), stay at home order for 14 days, schools closed.	80	8443	94.3
	2	20^th^ July—18^th^ October	12	Limitations on gatherings lifted, public places reopened.	49	2557	28.6
	3	19^th^ October—27^th^ December	10	Cases begin to rise in second wave. Low stringency lockdown, restrictions start to be implemented again mid-December.	26	5839	65.2
Kambia	0	1^st^ January—15^th^ March	11	Pre-COVID	0		
	1	16^th^ March—19 July	18	First wave of reported infections. Gatherings of over 100 banned, state of emergency (declared 31 March).	60	1711	214.5
	2	20^th^ July—27^t^ December	22	Low number of cases and low stringency lockdown measures.	33	849	106.4
Masaka	0	1^st^ January—15^th^ March	11	Pre-COVID	0		
	1	16^th^ March—20^th^ September	26	Gatherings restricted, schools closed, nationwide lockdown, transport restrictions.	83	6287	137.5
	2	21^st^ September—27^th^ December	13	Some public places re-opened, curfew remained in place.	59	27524	601.7

^1^ Each year was classified into 52 weeks and data was analysed by week, the dates included in each period varied slightly from year to year, dates provided in the table are those corresponding to the selected weeks in 2020, which defined the periods of analysis. The periods of analysis were selected based on COVID-19 case numbers and the implementation of lockdown measures.

### Data collection

Trained staff visited each of the selected facilities and collected general information from the head of each health facility, detailing: the number of staff members, the services provided and estimated catchment population, and any other known service disruptions over the study period. Weekly counts of the number of consultations in the registration book for each service from January 2018 to December 2020 were entered directly onto an electronic REDcap database on computer tablets [12, 13]. Data were collected on the weekly number of under-5 outpatient department (OPD) consultations, first antenatal care (ANC) visits, third doses of the diphtheria-tetanus-pertussis (DTP3) vaccine delivered, first doses of measles-containing vaccine (MCV1) delivered, first doses of human papillomavirus vaccine (HPV1) delivered, family planning (FP) consultations, new HIV diagnoses and HIV care visits (including for ART replenishment). If data were available on the probable diagnosis at the OPD visit, the numbers of children diagnosed with respiratory diseases and probable malaria were noted, and this was further broken down by whether the child was referred for admission or treated as an outpatient.

Qualitative data were collected with consent by trained social scientists in interviews and focus group discussions with 12–30 healthcare workers and 12–30 community members in each setting. Topic guides explored experiences providing and accessing care during the pandemic. These data inform the discussion of the data presented in this paper but are reported in full separately (Myfanwy James et al., article in print).

### Sample size estimation and statistical analysis

Assuming an average of 157 OPD visits per week, with a standard deviation of 73, a sample of at least 20 facilities per period enabled the study to detect a relative change of 30% with 80% power at the 5% significance level.

Distinct periods of the pandemic were defined using the reported ‘waves’ of COVID-19 using Ministry of Health (MOH) data in each area, reported lockdown measures, and the estimated ‘stringency’ of the lockdown in each setting [3] (Table 2). For each defined period of analysis, negative binomial regression was used to estimate the relative change in count data, comparing 2018, 2019 and 2020, using robust standard errors to adjust for autocorrelation and controlling for ‘month’ as a season parameter. Clinic level random effects were included to account for between clinic differences and within-clinic clustering of data. Climate data from the nearest available weather station were downloaded and assessed in the model as potential confounders including average daily highest temperature and average precipitation.

This study was approved by the Comite National d’Ethique de la Sante (CNES) of the DRC, the Sierra Leone Ethics and Scientific Review committee, the Uganda Virus Research Institute Research Ethics Committee (ref GC/127/21/04/821), the Uganda National Council for Science and Technology (ref HS1430ES), the London School of Hygiene and Tropical Medicine Ethics Committee (ref 22726) and local health authorities in each area. The requirement for informed consent was waived for the quantitative data collection reported here as individual level data were not collected.

## Results

The three settings were distinct in health service organisation and context. Facilities in Goma were relatively large, with an average catchment population of 30,000 and on average 12 registered/trainee nurses per facility in 2020. Masaka facilities served an average of 11,000 people and had an average of 4 registered/trainee nurses per facility in 2020. In Kambia, primary healthcare facilities were generally small, serving 5,000 people and were staffed by on average just 1 registered/ trainee nurse alongside supporting staff (community health volunteers, midwives etc; Table 3).

**Table 3:** Catchment populations and staffing over time in the selected facilities.

	Goma			Kambia			Masaka		
Facility characteristics	2018	2019	2020	2018	2019	2020	2018	2019	2020
**Catchment population**									
Mean per facility (s.d.)	28,048	29,137	30,011	4,493	5,113	5,078	10,076	9,622	11,399
Range per facility	9,820–52,733	10,115–54,315	10,418–55,944	2,759–7,400	1,168–17,724	1,259–19,318	1,652–25,877	1,686–26,415	1,721–50,381
Number of facilities with data	21	21	21	15	18	21	24	23	24
**Staff**									
Mean number of ***registered/ trainee nurses*** per facility (range)	10.5 (7–18)	11.2 (4–21)	11.9 (2–21)	~	~	0.7 (0–3)	~	~	3.8 (0–13)
Number of facilities with data	14	15	21	0	0	29	0	0	25

~: missing data; s.d.: standard deviation.

### Goma, DRC

Data were available from all 21 facilities in 2019 and 2020 for all services; some facilities were missing data in 2018 (S1 Fig in S1 File). Service activity levels per facility per week were highly variable (S2 Fig in S1 File). In January-March, point estimates indicate that service activity may have been increasing across the years (2018, 2019, 2020) for many services (S1 Table in S1 File). There were significantly higher numbers of consultations for OPD (PR 1.38 (95% confidence interval (CI) 1.09–1.74)), DTP3 (PR 1.21 (95%CI 1.07–1.36)) and ANC services (PR 1.26 (95%CI 1.07–1.49)) in 2020 compared to 2019. There was no evidence of a difference in the number of consultations for other services in this period.

**Fig 1:**
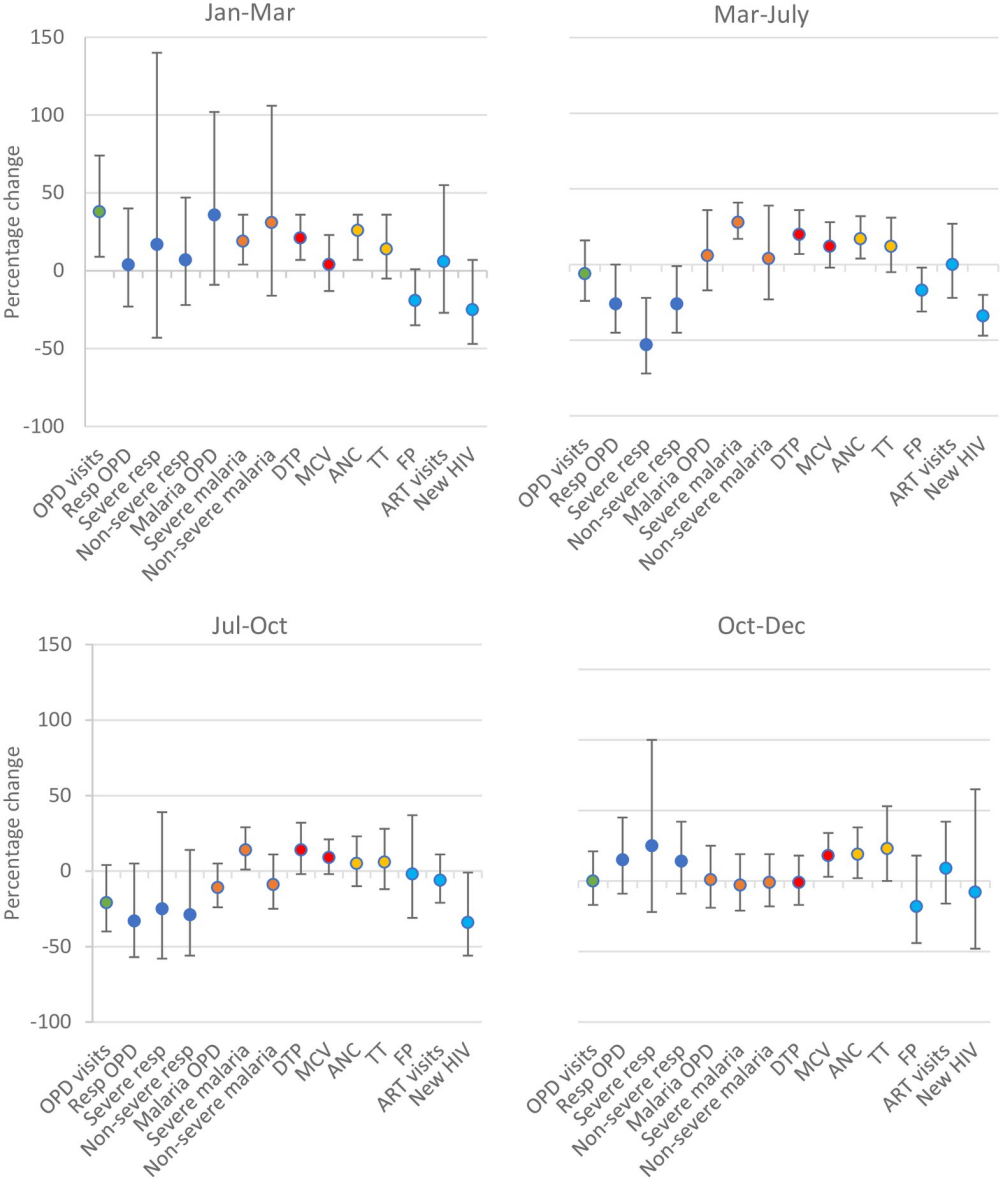
The percentage change in activity levels in 2020 compared to 2019, for each outcome, in each period, in Goma^1^. ^1^ The percentage change was calculated as (1-RR_2020/2019_)*100; the RRs for 2018 vs 2019 and 2019 vs 2020 are included in S1 Table (S1 File).

In period 1 of the pandemic (March-July), there was no evidence of a difference in the number of OPD consultations in 2020 compared to 2019 and this was sustained in periods 2 (July-October) and 3 (October-December; Fig 1). The number of DTP3 doses delivered in period 1 of the pandemic was 20% higher in 2020 compared to similar calendar months in 2019 (95%CI 7–36), but there was no evidence of a difference between 2020 and 2019 in periods 2 and 3. The point estimates for the difference in the number of MCV1 doses delivered indicated a 12–18% higher number of doses in 2020 compared to 2019; this was only statistically significant in period 3 of the pandemic. The number of first ANC visits in period 1 and 3 of the pandemic were 17% (95%CI 4–32%) and 19% (95%CI 2–38) higher than in the same periods in 2019; there was no evidence of a difference in period 2. There was no evidence of a difference in TT doses delivered, relative to the number delivered in 2019, in any of the calendar periods. There was evidence of 17% fewer FP consultations in period 1 of the pandemic (95%CI 2–31), but this difference disappeared in periods 2 and 3. The number of new diagnoses of HIV were 34% lower in both period 1 (95%CI 20–47) and period 2 (95%CI 1–56) of lockdown compared to the same periods the year before, but this effect disappeared in period 3. There was no evidence of a change in repeat ART replenishment visits, in any of the periods (Fig 1).

### Kambia, Sierra Leone

**Fig 2:**
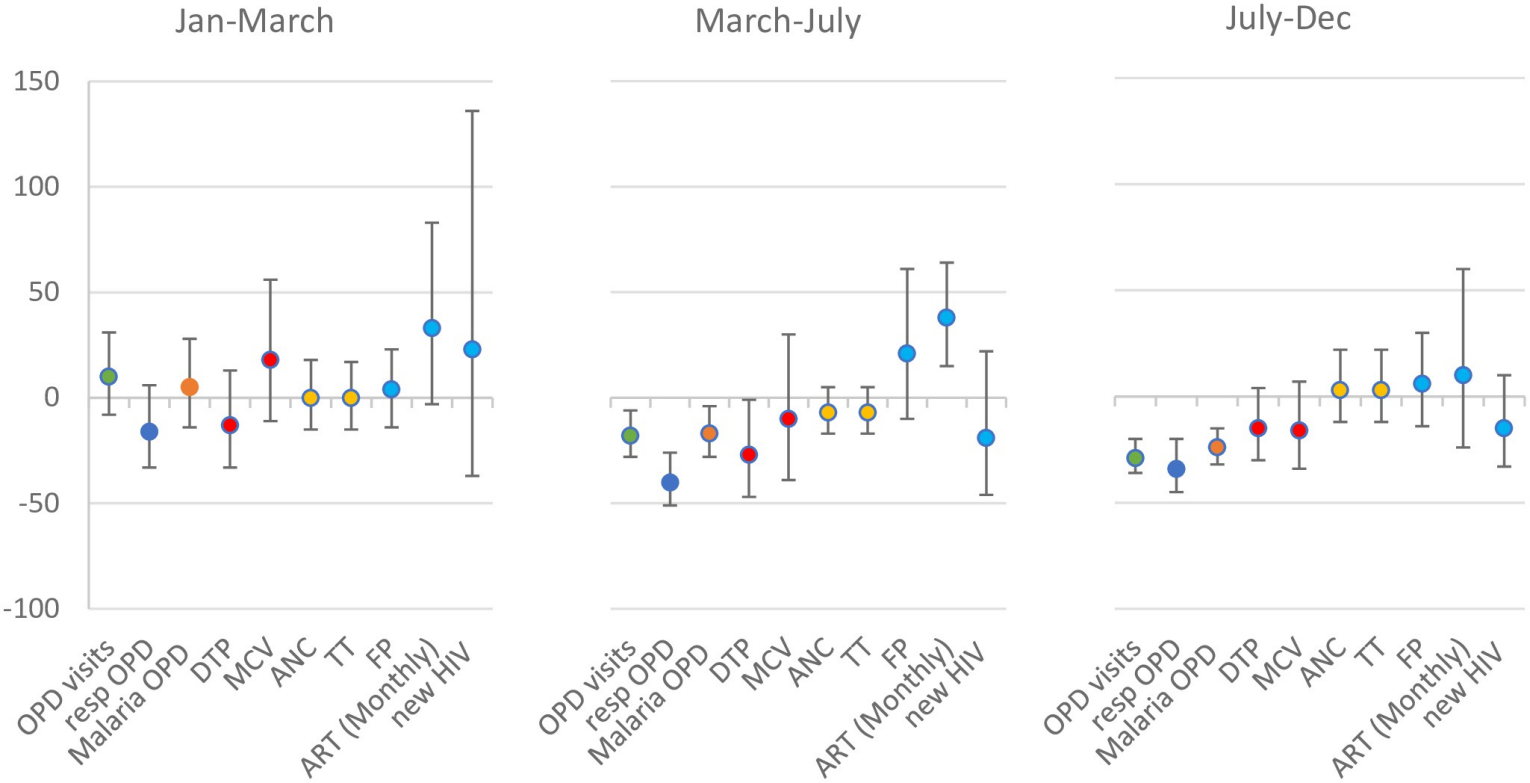
The percentage change in activity levels in 2020 compared to 2019, for each outcome, in each period, in Kambia, Sierra Leone^1^. ^1^ The percentage change was calculated as (1-RR_2020/2019_)*100; the RRs for 2018 vs 2019 and 2019 vs 2020 are included in S2 Table (S1 File).

Data were available from 25–29 facilities for all of the major service groups except HIV services (S3 Fig in S1 File); only 5–6 facilities had data on HIV services for 2018–2020. Data on whether outpatients were referred to a higher-level facility or treated as outpatients were incomplete and not analysable. Service activity levels per facility per week were highly variable (S4 Fig in S1 File). In January-March, there was no evidence of a difference in the level of activity for any of the services across any of the three years 2018, 2019 and 2020 (S2 Table in S1 File, Fig 2).

In period 1 of the pandemic in Sierra Leone (March-July), there were 18% fewer OPD consultations, compared to similar periods in 2019; this reduction was sustained in period 2 (July-December; 29% fewer OPD consultations (95%CI 20–36%)). When broken down by diagnosis, there were fewer respiratory and malaria OPD visits. There were 27% fewer DTP3 doses delivered in period 1 in 2020 compared to 2019 (95%CI 1–47%); but this difference disappeared in period 2. There was no evidence of a difference in the number of MCV1 doses delivered, ANC consultations, FP consultations or new HIV diagnoses in 2020 compared to 2019 in any of the periods of analysis, although CI are wide. In period 1 of the pandemic, there were 38% more repeat ART replenishment visits in 2020 compared to 2019, but this difference disappeared in period 2.

### Masaka, Uganda

**Fig 3:**
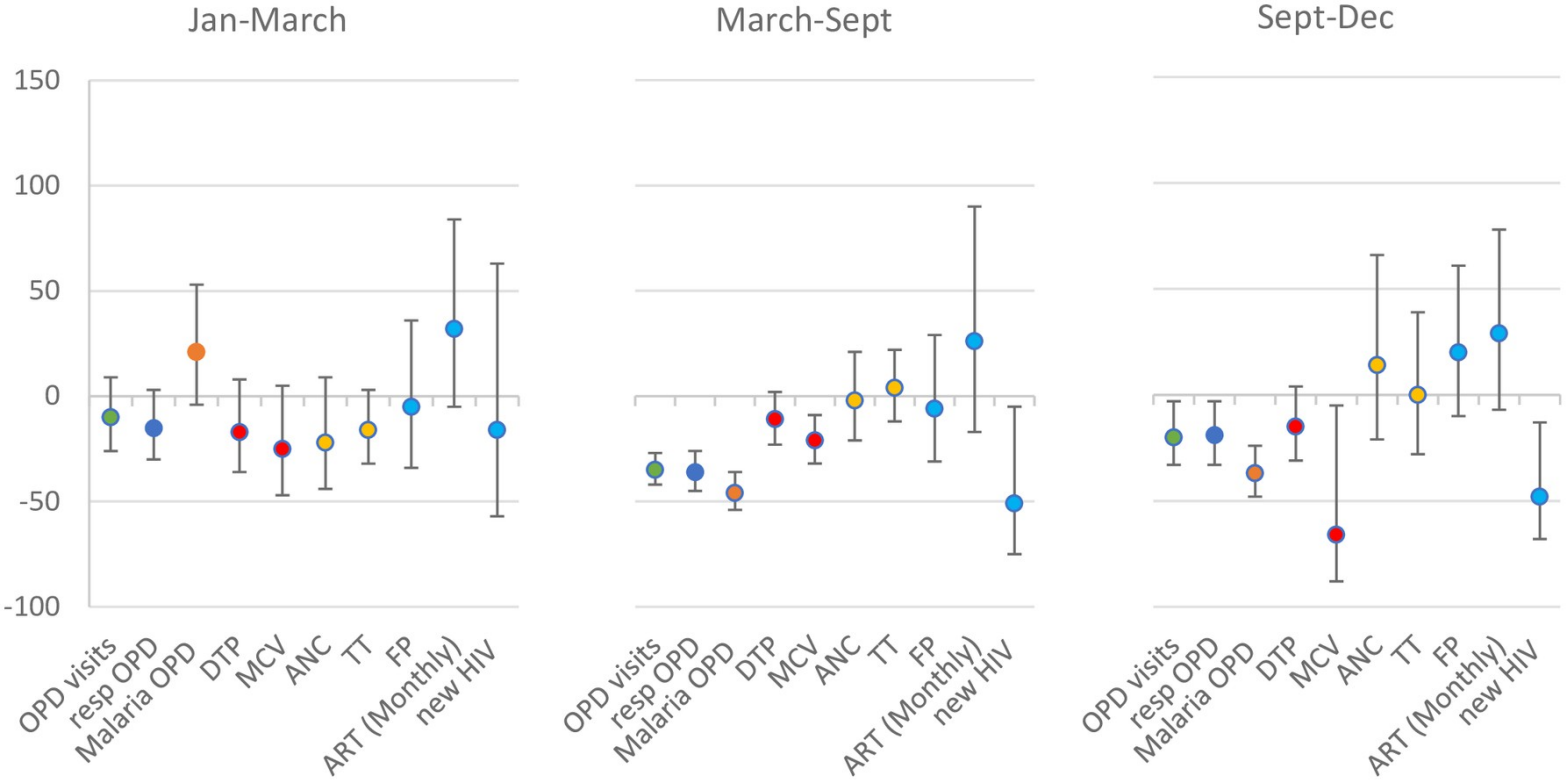
The percentage change in activity levels in 2020 compared to 2019, for each outcome, in each period, in Masaka, Uganda^1^.

Data were available from 20–25 facilities in 2019 and 2020 for most services; some facilities were missing data in 2018 (S5 Fig in S1 File). HIV service data were only available from 12 facilities in 2020, compared to 15–16 facilities in 2019. Mean counts of service activity per facility per week were highly variable (S6 Fig in S1 File). In January-March, there was no evidence of a difference in the level of activity for any of the services across any of the three years 2018, 2019 and 2020 (S3 Table in S1 File, Fig 3).

In period 1 of the pandemic in Uganda (March-September), there were 35% fewer OPD visits (95%CI 27–42) in 2020 compared to 2019, and this was sustained into period 2 (20% fewer OPD visits (95%CI 3–33; Fig 3)). In period 1 and 2 of the pandemic, point estimates indicate 11–15% fewer DTP3 doses delivered in 2020 compared to 2019, although confidence intervals cross the null. There were 21% fewer MCV1 doses delivered in period 1 of the pandemic in 2020 compared to 2019 (95%CI 9–32), and this difference increased in period 2 with 66% fewer MCV1 doses than in the previous year (95%CI 5–88). There was no evidence of a difference in the number of consultations for ANC, FP or ART replenishment when comparing 2020 and 2019 in any of the periods of analysis. The number of new HIV diagnoses in 2020 was half that of 2019, in both period 1 (51% reduction 95%CI 5–75) and period 2 (48% reduction 95%CI 13–68) of the pandemic.

Uganda was the only setting to deliver HPV vaccine in the study period. There were 77% fewer HPV vaccine doses delivered to girls aged 10 in period 1 of lockdown (March-September 2020), compared to 2019. There were substantially more doses delivered in period 2 of lockdown and overall, the number of first doses of HPV vaccine delivered in each year was similar.

## Discussion

In this analysis of weekly service activity levels across health facilities in Goma (DRC), Masaka (Uganda) and Kambia (Sierra Leone), we observed some reductions in primary care utilization/ provision during the lockdowns in 2020. The change in activity levels differed across services and settings (Table 4).

**Table 4:** Summary of percentage change in activity comparing 2020 with 2019 activity levels, for each period of lockdown by area.

Area	Goma^1^			Kambia^2^		Masaka^3^	
Service	Period 1	Period 2	Period 3	Period 1	Period 2	Period 1	Period 2
**OPD**	-6% (-24, +16)	-21% (-40, +4)	0 (-17, 21)	**-18% (-6, -28)**	**-29% (-20, -36)**	**-35% (-27, -42)**	**-20% (-33, -3)**
**DTP**	**+20 (+7, +36)**	+14% (-2, +32)	-1 (-17, 18)	**-27% (-1, -47)**	-15% (-30, +4)	-11% (+2, -42)	-15% (-31, +4)
**MCV**	+12% (-2, +28)	+9% (-2, +21)	**+18 (3, 34)**	-10% (-39, +30)	-16% (-34, +7)	**-21% (-9, -32)**	**-66% (-88, -5)**
**ANC**	**+17 (+4, +32)**	+5% (-10, +23)	**+19 (2, 38)**	-7% (-17, +5)	+3% (-12, +22)	-2 (+21, -21)	+14, (-21, +66)
**FP**	**-17% (-2, -31)**	-2% (-31, +37)	-18 (-44, 18)	+21% (-10, +61)	+6% (-14, +30)	-6 (+29, -31)	+20 (-10, +61)
**ART**	0% (-22, +27)	-6% (-21, +11)	+ 9 (-16, 42)	**+38% (+15, +64)**	+10% (-24, +60)	+26% (-17, +90)	+29 (-7, +78)
**new HIV**	**-34% (-20, -47)**	**-34% (-56, -1)**	-8 (-48, 65)	-19% (-46, +22)	-15% (-33, +10)	**-51% (-5, -75)**	**-48 (-68, -13)**
**HPV dose 1**	~	~	~	~	~	**-77% (-43, -81)**	+84% (-9, +372)

^1^ In Goma, Period 1: 23 Mar—19 Jul 2020, Period 2: 20 Jul—18 Oct 2020; Period 3: 19 Oct—27 Dec.

^2^ In Kambia, Period 1: 16 Mar—19 Jul 2020, Period 2: 20 July—27 Dec 2020.

^3^ In Masaka, Period 1: 16 Mar—20 Sept 2020, Period 2: 21 Sept—27 Dec 2020.

**In Goma, DRC**, we observed little to no difference in service activity levels in 2020 compared to 2019 and 2018. We only observed fewer family planning consultations, perhaps due to the discontinuation of the International Red Cross funding for family planning in 2020 (pers. Comm. P Obady). The reduction in HIV diagnoses reflects a reported stockout of diagnostic tests for HIV during the COVID 19 pandemic (pers. comm P Obady). Qualitative data reflected the perceived low risk of COVID-19 disease within a health system which was only just emerging from a recent Ebola epidemic [15], and past expertise in responding to outbreaks of public health concern. Temperature screening and infection prevention and control measures had been in place in primary health facilities for some time to screen for Ebola and the community were familiar with these measures [16]. Among health management, COVID-19 was perceived from the beginning as ‘not as bad as Ebola’. However, crisis response teams conducted substantial community engagement, delivering messages on preventative measures and the availability of health services. The lockdown within Goma eased relatively quickly and crucially, transport options remained available for people to get to health centres (qualitative data reported in full by Myfanwy James et al. article in press).

**In Kambia, Sierra Leone,** we observed some reductions in OPD and DTP3 activity levels during the pandemic. Qualitative data from community members described fears of getting infected at the health facility, forced vaccination and vaccine side-effects and fears of being diagnosed with COVID-19 and the potential consequences (including quarantine). Health care workers also reported reduced staffing at facilities. However, experiences during the Ebola epidemic of 2014–16 may have mitigated some of the impact of lockdown measures. Social mobilisation campaigns were reportedly held in the area alongside a national campaign to encourage the population to access routine healthcare [17]. We observed an increase in HIV ART replenishment visits as health communication messages at the time encouraged collection of a 3-month supply of ART due to uncertainties around service continuation at the beginning of the pandemic.

**In Masaka, Uganda**, we observed substantial reductions in service activity for several services including OPD, DTP3, MCV1, HIV care and HPV vaccination. The lockdown enforced during the pandemic was stringent and prolonged with reduced transport options [3]. Healthcare workers were reportedly out of station, sick, or quarantining, and one died (pers. Comm. F. Nakiyimba). Although the usual outreach for integrated mother and child health services continued and potentially mitigated the impact of the lockdown on FP, ANC and infant immunisation service uptake, there was reportedly no budget for additional outreach services e.g. for OPD and HIV care (pers. Comm. F. Nakiyimba). Our findings support another study documenting a 77% decrease in new HIV diagnoses in the first weeks of April 2020 compared to January-March 2020 [8]; additionally, we found that this decrease was sustained throughout 2020.

HPV vaccine is usually delivered in Uganda via school-based outreach programmes [18]. Although schools remained closed throughout 2020, the MOH supplied a specific grant to support HPV vaccine delivery through community outreach, integrated with child health days, later in 2020 (pers. Comm. F Nakiyimba). This mitigation strategy meant that, by the end of the year, the number of girls who had received their first dose of HPV was no different to previous years. MCV1 was given at these health days but uptake may have suffered from lack of mobilisation or awareness.

There are several limitations of this analysis, the before-after design meant that we could not account for secular trends and there is evidence to suggest increasing catchment populations and increasing service activity across the three years in Goma. The lack of difference we observed between the pandemic periods compared to pre-pandemic periods may have been a reduction compared to what would be expected if 2020 had been ‘a normal year’. We assumed catchment populations are relatively stable over time in order to compare utilisation across years with count data; however, some reports from Uganda indicate that there could have been population movement into Masaka from nearby cities in 2020, thus increasing the population, but this was transient and the effect was reported to last for only a few months [19, 20]. We assessed the comparability of the years of analysis with respect to climate factors and found no evidence of a difference in average maximum or minimum temperatures or atmospheric pressure between the years, by period. However, data were only available from international airport weather stations so do not account for local climate variations. We focused this analysis on government facilities so that recommendations were relevant to Ministry of Health officials; however, this means we do not have any evidence of whether the reductions in utilisation of government facilities coincided with an increase in the use of private or traditional health providers. We did not measure whether the vaccine doses were delivered via outreach or via routine services and therefore cannot estimate the extent to which service continuity relied on outreach during this time. The localised nature of the pandemic restrictions and the mitigation measures put in place makes it difficult to generalise our results to other areas of the same country or other settings.

## Conclusion

We observed some reductions in primary care utilization/ provision during the lockdowns in 2020 in three distinct settings. In Goma, DRC, family planning and HIV service activity levels decreased by 17–34% but other services documented an increase. In Kambia, Sierra Leone, outpatient and immunisation services decreased by 18–29% during the pandemic relative to previous years, but other services were unaffected. In Masaka, Uganda, outpatient, immunisation and HIV services decreased by 21–66% during the pandemic and HPV vaccine delivery was affected but bounced back with a campaign later in the year. The level of disruption appeared to correlate qualitatively with the strength of lockdown measures in the different settings and community attitudes towards the risk posed by COVID-19 especially in contexts with a history of Ebola outbreak responses. Mitigation strategies such as health communication campaigns and outreach services were reported as potential reasons why the impact of lockdowns on primary healthcare services was limited in some areas.

## Supporting information

S1 File (DOCX)

S2 File (DOCX)
