# Supplementary material for: The impact of the COVID-19 pandemic on the provision & utilisation of primary health care services in Goma, Democratic Republic of the Congo, Kambia district, Sierra Leone & Masaka district, Uganda
Source: PLoS One. 2023 Jun 2;18(6):e0286295. doi: 10.1371/journal.pone.0286295 (PMC10237403; doi:10.1371/journal.pone.0286295)
Supplement: S2 File — (DOCX) [file pone.0286295.s002.docx]

**L'impact de la pandémie de COVID-19 sur la fourniture et l'utilisation des services de soins de santé primaires à Goma, en République démocratique du Congo, dans le district de Kambia, en Sierra Leone et dans le district de Masaka, en Ouganda.**

K Kasonia^+1^ & D Tindanbil^+2^ , J Kitonsa^3^ , K Baisley^4^ , F Zalwango^3^ , L Enria^2^ , A Mansaray^2^ , M James^1^ , Y Nije^2^ , D Tetsa Tata^1^ , B J Lawal^2^ , A Drammeh^2^ , B Lowe^4^ , D Mukadi-Bamuleka^5^ , S Mounier-Jack^4^ , F Nakiyimba^6^ , P Obady^7^ , J Muhavi^7^ , J S Bangura^8,9^ , B Greenwood^4^ , M Samai^8^ , B Leigh^8^ , D Watson-Jones^+4,10^ & H Kavunga-Membo^+5^  & E Ruzagira^+3^ & K E. Gallagher^+4*^

**^+^ Contribution égale**

**Affiliations**

^1^ Partenariat de recherche LSHTM-INRB, Goma, République démocratique du Congo

^2^ Partenariat de recherche LSHTM-COMAHS, Kambia, Sierra Leone

^3^ Unité de recherche MRC/UVRI et LSHTM Ouganda, Entebbe, Ouganda

^4^ London School of Hygiene & Tropical Medicine (LSHTM), Londres, Royaume-Uni

^5^ Laboratoire Rodolphe Merieux-Institut national de recherche biomédicale (INRB), Goma, République démocratique du Congo

^6^ Ministère de la santé, district de Masaka, Ouganda

^7^ Ministère de la santé, Goma, République démocratique du Congo

^8^ Collège de médecine et des sciences paramédicales de l'Université de Sierra Leone (COMAHS), Freetown, Sierra Leone

^9^ Ministère de la santé, district de Kambia, Sierra Leone

^10^ Unité des essais d'intervention de Mwanza, Institut national de la recherche médicale, Mwanza, Tanzanie

***Auteur correspondant :** Katherine Gallagher, Département d'épidémiologie des maladies infectieuses, Faculté d'épidémiologie et de santé des populations, London School of Hygiene and Tropical Medicine, Keppel Street, Londres, WC1E 7HT, Royaume-Uni. Courriel : [Katherine.gallagher@lshtm.ac.uk](mailto:Katherine.gallagher@lshtm.ac.uk)

**Le nombre de mots du résumé : 299/300**

**Nombre de mots : 3837/ 5000**

**Mots clés : pandémie, soins primaires, covid, impact**

**Résumé**

**Introduction**

Cette étude visait à déterminer si la pandémie de COVID-19 avait un impact sur le nombre de personnes vues dans les établissements publics en Ouganda, en République démocratique du Congo (RDC) et en Sierra Leone pour des services essentiels de soins de santé primaires.

**Méthodes**

Le nombre de consultations hebdomadaires pour les soins prénatals (ANC), les consultations externes (OPD), le programme élargi de vaccination (PEV), les services de planification familiale (PF) et le VIH, pour la période de janvier 2018 à décembre 2020, a été collecté auprès de 25 établissements de soins de santé primaires du district de Masaka, en Ouganda, de 21 centres de santé à Goma, en RDC, et de 29 établissements du district de Kambia, en Sierra Leone. Des modèles de régression binomiale négative tenant compte du regroupement au niveau des établissements et de la saison ont été utilisés pour analyser les changements dans les niveaux d'activité entre 2018, 2019 et 2020.

**Résultats**

Nous n'avons trouvé aucune preuve que la pandémie de COVID-19 ait affecté le nombre de consultations OPD, PEV ou ANC à Goma. Les consultations de planification familiale ont été inférieures de 17% en mars-juillet 2020 par rapport à 2019, mais cette situation s'est rétablie en décembre 2020. Les nouveaux diagnostics de VIH ont été inférieurs de 34 % tout au long de 2020 par rapport à 2019. Par rapport aux mêmes périodes en 2019, les établissements de Sierra Leone ont eu 18 à 29 % de consultations OPD en moins tout au long de 2020, et 27 % de doses DTP3 en moins en mars-juillet 2020, mais cela s'est rétabli en juillet-décembre. Aucune différence n'a été constatée dans les autres services. En Ouganda, il y a eu 20 à 35 % de consultations en moins au bloc opératoire pour les enfants de moins de 5 ans, 21 à 66 % de doses de MCV1 en moins, et 48 à 51 % de nouveaux diagnostics de VIH en moins, tout au long de l'année 2020, par rapport à 2019. Il n'y a pas eu de différence dans le nombre de doses de VPH délivrées en 2020 par rapport à 2019.

**Conclusions**

Le niveau de perturbation semblait être en corrélation avec la force des mesures de confinement dans les différents contextes et les attitudes de la communauté vis-à-vis du risque posé par le COVID-19. Les stratégies d'atténuation telles que les campagnes de communication sur la santé et les services de proximité se sont avérées importantes pour limiter l'impact des mesures de confinement sur les services de soins de santé primaires.

**Messages clés**

**Ce que l'on sait déjà sur ce sujet** : La pandémie de COVID-19 et les mesures de réponse mises en place ont perturbé la fourniture et l'utilisation des services de soins de santé primaires dans le monde entier.

**Ce que cette étude apporte** : Nous documentons que la pandémie de COVID-19 a eu un impact varié sur différents services dans trois environnements distincts sur le continent africain. L'ampleur de l'impact de la pandémie sur les services est corrélée à la rigueur des mesures de confinement, à la perception par la communauté du niveau de danger posé par la pandémie et à l'exposition antérieure des communautés aux épidémies d'Ebola et aux mesures de réponse concomitantes.

**En quoi cette étude pourrait-elle influer sur la recherche, la pratique ou la politique** ? Des stratégies telles que les campagnes de communication et les services de proximité ont limité l'impact des fermetures sur les services essentiels et seraient des stratégies précieuses à mettre en œuvre lors de futures épidémies.

**Introductio**n

Les enquêtes pulsées de l'Organisation mondiale de la santé (OMS) ont indiqué que la pandémie de COVID-19 a affecté la fourniture et l'utilisation des services essentiels de soins de santé primaires dans > 90 % des pays du monde. Dans la première enquête, menée entre mai et juillet 2020, 25 services essentiels ont été évalués dans 105 pays. Presque tous les pays ont signalé un changement partiel (5 % à 50 %) ou grave (> 50 %) dans la fourniture ou l'utilisation des services. Les pays à revenu faible ou moyen inférieur ont été plus touchés que les pays à revenu plus élevé.(1). Lors de la deuxième enquête, menée entre janvier et mars 2021, 94 % des 135 pays ayant participé à l'enquête ont fait état de perturbations résiduelles des services(2). En réponse à la pandémie, diverses mesures de confinement telles que l'éloignement social, les fermetures, les couvre-feux, la fermeture des écoles et l'interdiction des rassemblements ont été mises en place dans le monde entier.(3). La réduction des moyens de transport et des revenus disponibles pendant les périodes de fermeture, ainsi que les craintes et les idées fausses concernant le risque et la possibilité d'accéder aux services, ont pu réduire l'utilisation des soins primaires. Les ressources de santé, notamment le personnel, les installations, les consommables, les traitements et les équipements de protection individuelle, ont été redéfinies pour lutter contre la maladie, des mesures de distanciation sociale ont été mises en place dans les établissements et de nombreux travailleurs sont tombés malades, ce qui a réduit la capacité à fournir des services de santé essentiels dans de nombreux contextes.(1).

Des analyses documentaires ont déjà montré que COVID-19 a eu un impact considérable sur les soins de santé primaires, tant au niveau des services que des patients.(4, 5)Cependant, presque toutes les études proviennent d'Europe ou des États-Unis. Le Royaume-Uni a signalé une réduction de 20 % des vaccinations contre la rougeole, trois semaines après l'annonce des mesures de distanciation sociale(6). Aux États-Unis, le moment de la pandémie a été corrélé avec une diminution significative du nombre de femmes arrivant dans un grand hôpital régional avec un plan de contraception.(7). Une méta-analyse de 14 études provenant de pays d'Europe, d'Asie et d'un pays d'Afrique a fait état d'une augmentation de 33 % du taux de mortinatalité pendant le confinement (IC à 95 % : 1,04, 1,69).(5). Il existe peu d'informations sur l'impact de la pandémie sur la fourniture et l'utilisation des services de soins de santé primaires dans les pays à revenu faible et intermédiaire (PRFM), notamment en Afrique. Une étude menée au Rwanda a mis en évidence une diminution significative de l'utilisation des soins prénatals, des accouchements en établissement, des soins postnatals et des vaccinations entre avril-mai 2020 et avril-mai 2019.(8). En Ouganda, une réduction de 75 % du dépistage du VIH et de l'initiation du traitement antirétroviral a été signalée au cours des trois premières semaines d'avril 2020 par rapport à la moyenne hebdomadaire pour la période de janvier à mars 2020.(9).

Le premier cas d'infection par le SRAS-CoV-2 en République démocratique du Congo (RDC) a été identifié le 10^th^ mars 2020. A partir du 18^th^ mars, en réponse à la pandémie, le gouvernement a mis en place des restrictions de mouvement, la fermeture des espaces publics, la limitation des rassemblements et le port obligatoire du masque en public (10). Le premier cas d'infection par le SRAS-CoV-2 en Sierra Leone a été enregistré le 30^th^ mars 2020.(11). Le président de la République de Sierra Leone a déclaré une urgence de santé publique pour douze mois et a imposé un couvre-feu du crépuscule à l'aube, des restrictions de mouvement, des interdictions de rassemblements publics, la fermeture des écoles et le port obligatoire de masques. Le premier cas d'infection par le SRAS-CoV-2 a été confirmé en Ouganda le 21^st^ mars 2020(12). Par la suite, le ministère de la Santé a imposé des restrictions de mouvement, la fermeture des espaces publics et des écoles, les rassemblements ont été limités et le port de masques a été rendu obligatoire en public. Toutes ces mesures ont pu affecter la prestation et l'utilisation efficaces des services de santé primaires dans ces pays.

Nous avons cherché à déterminer de quelle manière et dans quelle mesure la pandémie a eu un impact sur l'offre et l'utilisation des services de soins de santé primaires en 2020 dans trois contextes distincts avec différentes charges documentées de COVID-19 et différentes mesures de verrouillage. Nous nous concentrons sur le nombre de personnes vues dans les établissements de santé pour les soins prénataux, les services ambulatoires pour les moins de 5 ans, les vaccinations de routine, le planning familial et les services de traitement du VIH, en utilisant les données d'enregistrement sanitaire de routine. Des données qualitatives ont été collectées et éclairent la discussion des données présentées dans cet article mais sont rapportées séparément. Une traduction française de ce manuscrit est fournie dans les fichiers supplémentaires.

**Méthodes**

Cadre de l'étude et sélection des établissements de santé

L'étude a été menée dans trois zones : Goma, en République démocratique du Congo, le district de Kambia, au nord-ouest de la Sierra Leone, et le district de Masaka, au sud-ouest de l'Ouganda (**tableaux 1, 2**). Les autorités régionales et de district ont été contactées pour obtenir l'autorisation de mener l'étude et des listes complètes des établissements de santé de chaque région ont été compilées. Les établissements de santé privés ont été exclus de la sélection, car l'objectif du projet était d'informer la prestation de services de santé publique. Une sélection de centres de santé a été faite pour inclure les 25 centres de santé gouvernementaux disponibles à Masaka, les 21 centres de santé accessibles dans la zone urbaine de Goma ; et un générateur de nombres aléatoires a été utilisé pour sélectionner une sélection représentative de 29 établissements de santé à Kambia, proportionnellement au nombre total de postes de santé et de centres de santé dans le district.

Collecte des données

Le personnel formé a visité chacun des établissements sélectionnés et a recueilli des informations générales auprès du responsable de chaque établissement de santé, en détaillant : l'emplacement de l'établissement, le nombre de membres du personnel, les services fournis et l'estimation de la population desservie, ainsi que toute autre perturbation connue des services au cours de la période d'étude. Le personnel du projet a ensuite comptabilisé chaque semaine le nombre de consultations dans le registre d'enregistrement de chaque service de janvier 2018 à décembre 2020. Les données ont été saisies directement sur une base de données électronique REDcap sur des tablettes informatiques.(13, 14). Des données ont été collectées sur le nombre hebdomadaire de consultations dans les services de consultations externes (OPD) pour les moins de 5 ans, de premières visites de soins prénatals (ANC), de troisièmes doses de vaccin diphtérie-tétanos-coqueluche (DTP3) administrées, de premières doses de vaccin contenant la rougeole (MCV1) administrées, de premières doses de vaccin contre le papillomavirus humain (HPV1) administrées, de consultations de planification familiale (PF), de nouveaux diagnostics de VIH et de visites de soins du VIH (y compris pour le renouvellement du TAR). Si des données étaient disponibles sur le diagnostic probable lors de la visite à l'OPD, le nombre d'enfants diagnostiqués comme souffrant de maladies respiratoires et de paludisme probable était noté, puis ventilé selon que l'enfant était référé pour une admission ou traité en tant que patient externe.

**Tableau 1. Le cadre de l'étude et les centres de santé sélectionnés**

| **Pays** | **Région/ district sélectionné** | **Description et population totale** | **Total des établissements de santé publique dans la région** | **Installations sélectionnées pour la collecte de données quantitatives** | **Installations sélectionnées pour les entretiens qualitatifs** |
| --- | --- | --- | --- | --- | --- |
| **RDC** | Goma | Urbaine et suburbaine ; population estimée : 600 000 à 1 million | 39  (26 centres de santé, 13 hôpitaux de soins tertiaires) | 21  (21 centres de santé^1^ ) | 12 |
| **Sierrra Leone** | Kambia | Suburbaine et rurale ; population estimée : 350,000 | 68  (55 postes de santé, 15 centres de santé, 1 hôpital) | 29  (22 postes de santé, 6 centres de santé, 1 hôpital avec un département de soins de santé primaires.^2^ ) | 15 |
| **Ouganda** | Masaka | Rural ; population estimée : 307,000 | 26  (25 établissements de santé, 1 hôpital de référence) | 25  (14 centres de santé de niveau II, 9 de niveau III, 2 de niveau IV^3^ ) | 15 |

^1^ A Goma, les centres de santé fournissent des soins de santé primaires à la population urbaine ; 21 des 26 centres de santé publics ont été sélectionnés en raison de contraintes de sécurité et de logistique lors de la collecte des données.

^2^ À Kambia, les postes de santé, les centres de santé communautaires et certains hôpitaux fournissent des soins primaires à la population. Les postes de santé (postes de santé communautaires ou maternels et infantiles) sont de petite taille et fournissent des services dans des zones rurales éloignées ; les centres de santé communautaires sont plus grands, basés dans des endroits centraux au sein de chaque chefferie et peuvent disposer de laboratoires et de services de chaîne du froid plus cohérents. L'hôpital de district de Kambia a été inclus en tant que principal prestataire public de soins de santé primaires dans la ville de Kambia.

^3^ A Masaka, les établissements de santé de niveau II fournissent des services ambulatoires à la paroisse environnante, les établissements de niveau III sont des établissements de sous-comté légèrement plus grands qui peuvent avoir des maternités, et les établissements de niveau IV ont des services hospitaliers et ambulatoires.

Estimation de la taille de l'échantillon et analyse statistique

Les données du système d'information sur la gestion de la santé (HMIS) pour le district de Masaka, en Ouganda, ont été utilisées pour estimer la taille de l'échantillon nécessaire à l'étude pour détecter un changement relatif de 30 % en comparant une seule semaine en 2020 avec la même semaine l'année précédente. En supposant une moyenne de 157 visites OPD par semaine, avec un écart type de 73, un échantillon d'au moins 20 établissements par période permettrait à l'étude de détecter un changement relatif de 30% avec une puissance de 80% au niveau de signification de 5%.

La période d'analyse s'étendait du 1er^st^ janvier 2018 au 27^th^ décembre 2020. La disponibilité des données a été évaluée comme le nombre d'établissements avec des données pour chaque semaine d'analyse, ceci a été tracé dans le temps pour chaque zone, par service. Le nombre moyen de consultations par établissement disposant de données, par semaine, a été calculé et tracé dans le temps pour chaque service, dans chaque zone. La période pré-pandémique a été définie comme la période janvier-mars 2020 (**tableau 2**). Les différentes périodes de la pandémie ont été définies à l'aide des " vagues " de COVID-19 signalées par les données du ministère de la Santé dans chaque zone, des mesures de confinement signalées et de la " rigueur " estimée du confinement dans chaque établissement (Tableau 2).(3) (**Tableau 2)**. Pour chaque période d'analyse définie, une régression binomiale négative a été utilisée pour estimer le changement relatif du nombre, en comparant 2018, 2019 et 2020, en utilisant des erreurs standard robustes pour ajuster l'autocorrélation et en contrôlant le " mois " comme paramètre de saison. Des effets aléatoires au niveau de la clinique ont été inclus pour tenir compte des différences entre les cliniques et du regroupement des données au sein d'une même clinique. Les données climatiques de la station météorologique disponible la plus proche ont été téléchargées et évaluées dans le modèle comme un facteur de confusion potentiel.

**Tableau 2. Définition des périodes d'analyse basées sur la pandémie COVID et les mesures de confinement.**

| **Zone** | **Période** | **Dates approximatives de la période (2018-20) ^1^** | **N (semaines/an)** | **Description des mesures de confinement en place en 2020** | **Indice moyen de rigueur du confinement pour la période(3)** | **Nombre national de cas de COVID-19 signalés(15)** | **Nombre national de cas pour 1 million d'habitants** |
| --- | --- | --- | --- | --- | --- | --- | --- |
| Goma | 0 | 1^st^ janvier - 22^nd^ mars | 12 | Pré-COVID | 0 |  |  |
|  | 1 | 23 mars - 19^th^ juillet | 17 | La première vague de cas signalés atteint son apogée. État d'urgence (déclaré le 18^th^ mars), ordre de rester chez soi pendant 14 jours, écoles fermées. | 80 | 8443 | 94.3 |
|  | 2 | 20^th^ juillet - 18^th^ octobre | 12 | Les restrictions sur les rassemblements sont levées, les lieux publics sont rouverts. | 49 | 2557 | 28.6 |
|  | 3 | 19^th^ octobre - 27^th^ décembre | 10 | Les cas commencent à augmenter dans la deuxième vague. Les restrictions commencent à être appliquées à nouveau à la mi-décembre. | 26 | 5839 | 65.2 |
| Kambia | 0 | 1^st^ janvier - 15^th^ mars | 11 | Pré-COVID | 0 |  |  |
|  | 1 | 16^th^ mars - 19 juillet | 18 | Première vague d'infections signalées. Rassemblements de plus de 100 personnes interdits, état d'urgence (déclaré le 31 mars). | 60 | 1711 | 214.5 |
|  | 2 | 20^th^ juillet - 27^t^ décembre | 22 | Faible nombre de cas et mesures de confinement peu rigoureuses. | 33 | 849 | 106.4 |
| Masaka | 0 | 1^st^ janvier - 15^th^ mars | 11 | Pré-COVID | 0 |  |  |
|  | 1 | 16^th^ mars - 20^th^ septembre | 26 | Rassemblements restreints, écoles fermées, confinement national, restrictions des transports. | 83 | 6287 | 137.5 |
|  | 2 | 21^st^ septembre - 27^th^ décembre | 13 | Certains lieux publics ont rouvert, le couvre-feu est resté en place. | 59 | 27524 | 601.7 |

^1^ Chaque année a été classée en 52 semaines et les données ont été analysées par semaine, les dates incluses dans chaque période varient légèrement d'une année à l'autre, les dates fournies dans le tableau sont celles correspondant aux semaines sélectionnées en 2020, qui ont défini les périodes d'analyse. Les périodes d'analyse ont été sélectionnées en fonction du nombre de cas de COVID-19 et de la mise en œuvre des mesures de confinement.

Participation des patients et du public

Les autorités sanitaires locales ont été impliquées dans la conceptualisation et la mise en œuvre de l'étude ; le public n'a pas été impliqué dans la conceptualisation ou la conception de l'étude. Cette étude a été approuvée par le Comité national d'éthique de la santé (CNES) de la RDC, le comité d'éthique et d'examen scientifique de la Sierra Leone, le comité d'éthique de la recherche de l'Institut de recherche sur les virus de l'Ouganda, le Conseil national ougandais pour la science et la technologie, le comité d'éthique de la London School of Hygiene and Tropical Medicine et les autorités sanitaires locales de chaque région. Aucun consentement éclairé n'a été requis pour cette étude car les données au niveau individuel n'ont pas été collectées.

**Résultats**

Les trois sites se distinguent par l'organisation et le contexte des services de santé. Les établissements de Goma étaient relativement grands, avec une population moyenne de 30 000 personnes et en moyenne 12 infirmières diplômées/stagiaires par établissement en 2020. Les établissements de Masaka desservaient en moyenne 11 000 personnes et comptaient en moyenne 4 infirmières diplômées/stagiaires par établissement en 2020. À Kambia, les établissements de soins de santé primaires étaient généralement de petite taille, desservant 5 000 personnes et comptant en moyenne un seul infirmier diplômé/stagiaire, ainsi que du personnel de soutien (bénévoles en santé communautaire, sages-femmes, etc. ; **tableau 3**).

**Tableau 3. Populations desservies et effectifs au fil du temps dans les établissements sélectionnés**

|  | | **Goma** | | | | **Kambia** | | | **Masaka** | | | |
| --- | --- | --- | --- | --- | --- | --- | --- | --- | --- | --- | --- | --- |
| **Caractéristiques de l'installation** | | **2018** | | **2019** | **2020** | **2018** | **2019** | **2020** | **2018** | **2019** | **2020** |  |
| **Population du bassin versant** | |  | |  |  |  |  |  |  |  |  |  |
|  | Moyenne par établissement (s.d.) | | 28,048 | 29,137 | 30,011 | 4,493 | 5,113 | 5,078 | 10,076 | 9,622 | 11,399 |  |
|  | Gamme par installation | | 9,820- 52,733 | 10,115 - 54,315 | 10,418 - 55,944 | 2,759-7,400 | 1,168 - 17,724 | 1,259- 19,318 | 1,652- 25,877 | 1,686- 26,415 | 1,721- 50,381 |  |
|  | Nombre d'installations disposant de données | | 21 | 21 | 21 | 15 | 18 | 21 | 24 | 23 | 24 |  |
| **Personnel** | |  | |  |  |  |  |  |  |  |  |  |
|  | Nombre moyen d'***infirmières diplômées/stagiaires*** par établissement (fourchette) | | 10.5 (7-18) | 11.2  (4-21) | 11.9  (2-21) | ~ | ~ | 0.7  (0-3) | ~ | ~ | 3.8  (0-13) |  |
|  | Nombre d'installations disposant de données | | 14 | 15 | 21 | 0 | 0 | 29 | 0 | 0 | 25 |  |

~ : données manquantes ; s.d. : écart-type

Goma, RDC

Les données étaient disponibles dans les 21 installations en 2019 et 2020 pour tous les services ; certaines installations manquaient de données pour certains services en 2018 (**figure supplémentaire 1**). Les comptages moyens de l'activité de service par établissement et par semaine étaient très variables pour toutes les périodes et toutes les années (figure **supplémentaire 2**). Dans la période calendaire de janvier-mars, les estimations ponctuelles indiquent que l'activité de service pourrait avoir augmenté au fil des années (2018, 2019, 2020) pour de nombreux services (**tableau supplémentaire 1**). Il y avait un nombre significativement plus élevé de consultations pour les services OPD (risque relatif (RR) 1,38 (intervalle de confiance à 95 % (IC) 1,09-1,74)), DTP3 (RR 1,21 (IC à 95 % 1,07-1,36) et ANC (RR 1,26 (IC à 95 % 1,07-1,49) en 2020 par rapport à 2019. Il n'y avait aucune preuve d'une différence entre 2020 et 2019 dans le nombre de consultations pour d'autres services au cours de cette période.

Dans la période 1 de la pandémie (mars-juillet), il n'y avait aucune preuve d'une différence dans le nombre de consultations OPD en 2020 par rapport à 2019 et cela s'est maintenu dans les périodes 2 (juillet-octobre) et 3 (octobre-décembre ; **Figure 1**). Il y avait quelques indications que les visites à l'OPD pour des plaintes respiratoires avaient diminué, mais les visites à l'OPD pour le paludisme avaient augmenté dans la période 1 du lockdown, par rapport aux périodes similaires en 2018 et 2019, mais cette différence a disparu dans les périodes 2 et 3. Le nombre de doses de DTC3 délivrées au cours de la période 1 de la pandémie était 20 % plus élevé en 2020 par rapport aux mois calendaires similaires de 2019 (IC95% 7-36), mais rien n'indiquait une différence entre 2020 et 2019 dans les périodes 2 et 3. Les estimations ponctuelles de la différence du nombre de doses de MCV1 administrées indiquaient un nombre de doses supérieur de 12 à 18 % en 2020 par rapport à 2019 ; cette différence n'était statistiquement significative que pour la période 3 de la pandémie. Le nombre de premières consultations prénatales au cours des périodes 1 et 3 de la pandémie était supérieur de 17% (95%CI 4-32%) et 19% (95%CI 2-38) à celui des mêmes périodes en 2019 ; aucune différence n'a été mise en évidence pour la période 2. Il n'y avait aucune preuve d'une différence dans les doses de TT délivrées, par rapport au nombre délivré en 2019, dans aucune des périodes calendaires. Il y avait des preuves d'une diminution de 17% des consultations de PF au cours de la période 1 de la pandémie (95%CI 2-31), mais cette différence a disparu au cours des périodes 2 et 3. Le nombre de nouveaux diagnostics de VIH était inférieur de 34% au cours de la période 1 (95%CI 20-47) et de la période 2 (95%CI 1-56) du lockdown par rapport aux mêmes périodes l'année précédente, mais cet effet a disparu au cours de la période 3. Il n'y avait aucune preuve d'un changement dans les visites répétées de réapprovisionnement en ART, dans aucune des périodes (**Figure 1**).

Kambia, Sierra Leone

Des données étaient disponibles dans 25 à 29 établissements pour tous les principaux groupes de services, à l'exception des services liés au VIH (**figure supplémentaire 3**) ; seuls 5 à 6 établissements disposaient de données sur les services liés au VIH pour 2018-2020. Les données indiquant si les patients en consultation externe étaient orientés vers un établissement de niveau supérieur ou traités en tant que patients externes étaient incomplètes et non analysables. Les décomptes moyens de l'activité des services par établissement et par semaine étaient très variables pour toutes les périodes et toutes les années (**figure supplémentaire 4**). Au cours de la période prépandémique (janvier-mars), rien n'indique une différence dans le niveau d'activité de l'un ou l'autre des services au cours des trois années 2018, 2019 et 2020 (**tableau supplémentaire 2, figure 2**).

Au cours de la période 1 de la pandémie en Sierra Leone (mars-juillet), il y a eu 18 % de consultations OPD en moins, par rapport aux périodes similaires en 2019 ; cette réduction s'est maintenue au cours de la période 2 (juillet-décembre) où il y a eu 29 % de consultations OPD en moins (95 % IC 20-36 %) par rapport à la même période en 2019. En ventilant par diagnostic, il y avait moins de consultations OPD respiratoires et de paludisme. Il y a eu 27% de doses de DTC3 délivrées en moins au cours de la période 1 en 2020 par rapport à 2019 (95%CI 1-47%) ; mais cette différence a disparu au cours de la période 2. Il n'y avait aucune preuve d'une différence dans le nombre de doses de MCV1 délivrées, de consultations de CPN, de consultations de PF ou de nouveaux diagnostics de VIH en 2020 par rapport à 2019 dans aucune des périodes d'analyse, bien que les IC soient larges. Au cours de la période 1 de la pandémie, il y a eu 38% de visites de réapprovisionnement en TAR en plus en 2020 par rapport à 2019, mais cette différence a disparu au cours de la période 2.

Masaka, Ouganda

Des données étaient disponibles auprès de 20-25 établissements en 2019 et 2020 pour la plupart des services ; certains établissements manquaient de données pour certains services en 2018 (**figure supplémentaire 5)**. Les données relatives aux services VIH n'étaient disponibles que dans 12 établissements en 2020, contre 15-16 établissements en 2019. Le nombre moyen d'activités de services par établissement et par semaine était très variable pour toutes les périodes et toutes les années (**figure supplémentaire 6**). Au cours de la période pré-pandémique (janvier-mars), rien n'indique une différence dans le niveau d'activité de l'un ou l'autre des services au cours des trois années 2018, 2019 et 2020 (**tableau supplémentaire 3, figure 3**).

Au cours de la période 1 de la pandémie en Ouganda (mars-septembre), il y a eu 35 % de visites de consultation en moins (IC95 % 27-42) en 2020 par rapport à 2019, et cette tendance s'est maintenue au cours de la période 2 (septembre-décembre) avec 20 % de visites de consultation en moins (IC95 % 3-33 ; **Figure 3**) en 2020 par rapport à 2019. Au cours des périodes 1 et 2 de la pandémie, les estimations ponctuelles indiquent une diminution de 11 à 15 % des doses de DTC3 délivrées en 2020 par rapport à 2019, bien que les intervalles de confiance croisent le zéro. Il y a eu 21% de doses de MCV1 en moins dans la période 1 de la pandémie en 2020 par rapport à 2019 (95%CI 9-32), et cette différence a augmenté dans la période 2, avec 66% de doses de MCV1 en moins que l'année précédente (95%CI 5-88). Il n'y avait aucune preuve d'une différence dans le nombre de consultations pour la CPN, la PF ou la reconstitution du TAR en comparant l'activité des services de 2020 et de 2019 dans aucune des périodes d'analyse. Le nombre de nouveaux diagnostics de VIH en 2020 était deux fois moins élevé qu'en 2019, à la fois dans la période 1 (réduction de 51%, 95%CI 5-75) et dans la période 2 (réduction de 48%, 95%CI 13-68) de la pandémie.

L'Ouganda a été le seul cadre à délivrer le vaccin contre le VPH au cours de la période d'étude. Le nombre de doses de vaccin contre le VPH administrées aux filles âgées de 10 ans a diminué de 77 % au cours de la période 1 de verrouillage (mars-septembre 2020), par rapport à 2019. Cela avait rebondi avec une augmentation substantielle des doses dans la période 2 de verrouillage. Dans l'ensemble, le nombre de premières doses de vaccin contre le VPH administrées chaque année était similaire.

**Discussion**

Dans cette analyse des niveaux d'activité hebdomadaire des services dans les établissements de santé de Goma (RDC), Masaka (Ouganda) et Kambia (Sierra Leone), nous avons observé certaines réductions de l'utilisation/de la prestation des soins primaires pendant les lockdowns de 2020. Le changement des niveaux d'activité différait selon les services et les établissements (**tableau 4**).

**Tableau 4. Résumé de la variation en pourcentage de l'activité en comparant les niveaux d'activité de 2020 et de 2019, pour chaque période de confinement par zone**

| **Zone** | **Goma^1^** | | | **Kambia^2^** | | | **Masaka^3^** | | |
| --- | --- | --- | --- | --- | --- | --- | --- | --- | --- |
| **Service** | **Période 1** | **Période 2** | **Période 3** | **Période 1** | **Période 2** | **Période 1** | | **Période 2** |  |
| **OPD** | -6% (-24, +16) | -21% (-40, +4) | 0 (-17, 21) | -18% (-6, -28) | -29% (-20, -36) | -35% (-27, -42) | | -20% (-33, -3) |  |
| **DTP** | +20 (+7, +36) | +14% (-2, +32) | -1 (-17, 18) | -27% (-1, -47) | -15% (-30, +4) | -11% ((+2, -42) | | -15% (-31, +4) |  |
| **MCV** | +12% (-2, +28) | +9% (-2, +21) | +18 (3, 34) | -10% (-39, +30) | -16% (-34, +7) | -21% (-9, -32) | | -66% (-88, -5) |  |
| **ANC** | +17 (+4, +32) | +5% (-10, +23) | +19 (2, 38) | -7% (-17, +5) | +3% (-12, +22) | -2 (+21, -21) | | +14, (-21, +66) |  |
| **FP** | -17% (-2, -31) | -2% (-31, +37) | -18 (-44, 18) | +21% (-10, +61) | +6% (-14, +30) | -6 (+29, -31) | | +20 (-10, +61) |  |
| **ART** | 0% (-22, +27) | -6% (-21, +11) | + 9 (-16, 42) | +38% (+15, +64) | +10% (-24, +60) | +26% (-17, +90) | | +29 (-7, +78) |  |
| **nouveau VIH** | -34% (-20, -47) | -34% (-56, -1) | -8 (-48, 65) | -19% (-46, +22) | -15% (-33, +10) | -51% (-5, -75) | | -48 (-68, -13) |  |
| **HPV dose 1** | ~ | ~ | ~ | ~ | ~ | -77% (-43, -81) | | +84% (-9, +372) |  |

^1^ A Goma, période 1 : 23 mars - 19 juillet 2020, période 2 : 20 juillet - 18 octobre 2020 ; période 3 : 19 octobre - 27 décembre.

^2^ A Kambia, période 1 : 16 mars - 19 juillet 2020, période 2 : 20 juillet - 27 décembre 2020.

^3^ A Masaka, période 1 : 16 mars - 20 septembre 2020, période 2 : 21 septembre - 27 décembre 2020.

**À Goma, en RDC**, nous avons observé peu ou pas de différence dans les niveaux d'activité des services en 2020 par rapport à 2019 et 2018. Il y a eu un nombre plus élevé de vaccinations délivrées dans les périodes de pandémie de 2020 par rapport à 2019. Nous avons observé moins de consultations de planification familiale, ce qui est susceptible de refléter un véritable besoin non satisfait, car la Croix-Rouge internationale a cessé de financer la planification familiale à Goma en 2020 (comm. pers. P Obady). La réduction des diagnostics du VIH reflète une rupture de stock des tests de diagnostic du VIH pendant la pandémie du COVID 19. L'impact limité de la pandémie sur l'utilisation ou la fourniture de soins de santé primaires de routine était surprenant, étant donné que les données qualitatives fournies par les travailleurs de la santé décrivaient une réduction du personnel et des services perturbés pendant cette période (M. James et al. *manuscrit en préparation*). Les données qualitatives fournies par les membres de la communauté ont suggéré une certaine méfiance de leur part et la crainte que s'ils se rendaient dans les établissements, ils pourraient être mis en quarantaine de force, infectés par le COVID-19 ou recevoir le vaccin (F. Zalwango et al. *manuscrit en préparation*). Cependant, nos résultats peuvent s'expliquer par d'autres données qualitatives reflétant le faible risque perçu de la maladie COVID-19 au sein d'un système de santé qui sortait tout juste d'une récente épidémie d'Ebola.(16)et l'expertise passée dans la réponse aux épidémies de santé publique. Le dépistage de la température et les mesures de prévention et de contrôle de l'infection étaient en place dans les établissements de santé primaire depuis un certain temps pour dépister Ebola et la communauté était familière avec ces mesures. Parmi les responsables de la santé, le COVID-19 a été perçu dès le début comme " moins grave qu'Ebola ". Cependant, les équipes de réponse à la crise ont mené un important travail d'engagement communautaire, diffusant des messages sur les mesures préventives et la disponibilité des services de santé. Les campagnes de vaccination contre le choléra, les chefs religieux et les associations de femmes ont tous été ciblés par des messages éducatifs sur le COVID-19 et les services disponibles au centre de santé. De plus, le confinement à Goma était strict au départ, mais il s'est relâché relativement vite et a été respecté de manière variable ; de manière cruciale, les options de transport sont restées disponibles pour que les gens puissent se rendre dans les centres de santé.

**À Kambia, en Sierra Leone,** nous avons observé certaines réductions des niveaux d'activité OPD et DTP3 pendant la pandémie, avec environ 20 % de consultations OPD en moins et 30 % de doses DTP3 délivrées en moins en mars-juillet 2020 par rapport à la même période en 2019. Les données qualitatives des membres de la communauté ont décrit la peur d'être infecté dans l'établissement de santé, la vaccination forcée et les effets secondaires du vaccin, ainsi que la peur d'être diagnostiqué avec le COVID-19 et les conséquences potentielles (y compris la quarantaine). Les travailleurs de la santé ont également signalé une réduction du personnel dans les établissements. Cependant, les expériences vécues lors de l'épidémie d'Ebola de 2014-16 ont pu atténuer une partie de l'impact des mesures de confinement. Des campagnes de mobilisation sociale ont été organisées pour encourager la population à accéder aux soins de santé si elle en avait besoin pour les tests COVID, les vaccins et les services de routine. Nous avons observé une augmentation des visites de réapprovisionnement en TAR du VIH, potentiellement expliquée par les messages de communication sanitaire de l'époque qui encourageaient les personnes vivant avec le VIH à se rendre dans l'établissement pour récupérer une réserve de TAR de 3 mois en raison des incertitudes concernant la poursuite des services au début de la pandémie.

**À Masaka, en Ouganda**, nous avons observé des réductions substantielles de l'activité de plusieurs services, notamment les consultations externes, le DTC3, le MCV1, les soins VIH et la vaccination contre le VPH. Le confinement imposé pendant la pandémie était rigoureux et prolongé, avec des options de transport réduites et un couvre-feu de 19h00 à 06h30. Les services de santé ont été soumis à des tensions et des pressions considérables pendant la pandémie. Le personnel de santé a été absent pour cause de maladie et de quarantaine et l'un d'entre eux serait décédé. Bien que les services de proximité habituels pour les services intégrés de santé maternelle et infantile aient été maintenus et aient potentiellement atténué l'impact du confinement sur le recours aux services de PF, de CPN et de vaccination des nourrissons, il n'y avait initialement aucun budget supplémentaire pour des services de proximité supplémentaires, par exemple pour les consultations externes et les soins VIH, car on n'avait pas prévu qu'ils seraient nécessaires. Nos résultats sont soutenus par une autre étude qui documente une diminution de 77% des nouveaux diagnostics de VIH dans les premières semaines d'avril 2020 par rapport à la période janvier-mars 2020.(9)En plus de cette étude précédente, nous avons constaté que cette diminution s'est maintenue tout au long de l'année 2020.

En Ouganda, le vaccin anti-papillomavirus est généralement administré par le biais de programmes de sensibilisation en milieu scolaire ; bien que les écoles soient restées fermées tout au long de l'année 2020, le ministère de la Santé a fourni une subvention spécifique pour soutenir l'administration du vaccin anti-papillomavirus par le biais de programmes de sensibilisation communautaires, intégrés aux journées de santé infantile, plus tard en 2020. Cette stratégie d'atténuation a fonctionné et, à la fin de l'année, le nombre de filles ayant reçu leur première dose de VPH n'était pas différent de celui des années précédentes. Le vaccin MCV1 a été administré lors de ces journées de santé, mais la prise en charge a peut-être souffert d'un manque de mobilisation ou de sensibilisation, car l'offre était nettement inférieure en 2020 par rapport à 2019. Des campagnes de rattrapage de la rougeole seraient recommandées dans ce contexte pour éviter les épidémies.

Les données que nous rapportons sont conformes à une analyse mondiale qui fait état d'une diminution de 9 % des doses de DTC3 et de 10 % des doses de rougeole délivrées dans la région OMS AFRO en avril 2020.(17)une diminution plus faible que dans les autres régions du monde. Le recours à des programmes de proximité pendant les périodes de prestation de services de santé de routine, pour obtenir une couverture vaccinale élevée dans de nombreux contextes, peut avoir atténué l'impact de la pandémie. Le niveau de perturbation des services est également susceptible d'être très spécifique au contexte et de dépendre des relations locales entre le système de santé et la communauté, de la gestion du système de santé local et du budget disponible pour mener des activités supplémentaires afin d'atténuer l'impact. Une étude réalisée à Kinshasa a révélé une réduction de la couverture vaccinale contre la rougeole dans 2 des 4 zones de santé, les grands hôpitaux étant plus touchés que les centres de santé, mais dans l'ensemble, il n'y avait pas de différence dans le nombre de vaccins administrés. (18). Nous n'avons trouvé aucune preuve d'un impact sur les doses de vaccin contre la rougeole administrées à Goma. En Sierra Leone, les données de deux établissements de la région de Bo ont révélé une baisse de 32% du nombre de consultations prénatales.(19)tandis que les données de la région de Kambia n'ont montré aucun changement dans les consultations prénatales. Les centres de référence pour le paludisme de diverses régions de l'Ouganda n'ont enregistré aucun impact sur les visites de consultation pour le paludisme.(20)Ce résultat contraste avec la réduction de 20 à 30 % des consultations pour paludisme observée à Masaka. L'un des points forts de cette analyse est que nous avons concentré nos ressources sur la compréhension de l'utilisation et de la fourniture de services dans des contextes distincts et que nous avions la puissance statistique nécessaire pour détecter les effets éventuels.

Il y a plusieurs limites à cette analyse, la conception avant-après signifie que nous ne pouvions pas tenir compte des tendances séculaires et il y a des preuves qui suggèrent une augmentation des populations desservies et une augmentation de l'activité des services au cours des trois années à Goma. Le manque de différence que nous avons observé entre les périodes de pandémie et les périodes pré-pandémiques peut être une réduction par rapport à ce qui aurait été attendu si 2020 avait été " une année normale ". Cependant, nous n'avons pas eu la preuve d'une augmentation des populations du bassin versant ou d'une augmentation des niveaux d'activité au fil du temps pour Masaka ou Kambia. Nous avons évalué la comparabilité des années d'analyse en ce qui concerne les facteurs climatiques et n'avons trouvé aucune preuve d'une différence dans les températures moyennes maximales ou minimales ou dans la pression atmosphérique entre les années, par période. Cependant, les données n'étaient disponibles qu'à partir des stations météorologiques des aéroports internationaux et ne tiennent donc pas compte des variations climatiques locales. Nous avons concentré cette analyse sur les installations gouvernementales afin que les recommandations soient pertinentes pour les responsables du Ministère de la Santé ; cependant, cela signifie que nous n'avons aucune preuve que la réduction de l'utilisation des installations gouvernementales a coïncidé avec une augmentation de l'utilisation des prestataires de santé privés ou traditionnels. Les données qualitatives en RDC suggèrent que la population a pu choisir de se rendre dans les pharmacies, chez les tradipraticiens ou dans les structures de santé privées en pensant que le test COVID de l'Etat et les exigences de quarantaine qui en découlent, seraient moins strictement appliqués.

Nous n'avons pas déterminé si les doses de vaccin ont été administrées par le biais des services de proximité ou des services de routine. Nous ne pouvons donc pas estimer dans quelle mesure la continuité des services s'est appuyée sur les services de proximité pendant cette période, ni faire de recommandations sur la poursuite de ces services dans ces contextes, bien que les parties prenantes de la gestion de la santé en Ouganda suggèrent que les services de proximité ont atténué une partie de l'impact de l'interruption des services de routine. Nous supposons que les populations desservies sont relativement stables dans le temps afin de comparer l'utilisation entre les années avec les données de comptage. Nous disposons de peu de données sur les mouvements de population à l'intérieur et à l'extérieur de nos zones d'étude pendant la période de l'étude ; cependant, certains rapports en provenance de l'Ouganda indiquent qu'il pourrait y avoir eu un mouvement de population vers Masaka en provenance des villes voisines en 2020, augmentant ainsi la population, mais ce mouvement était transitoire et l'effet n'a duré que quelques mois. (21, 22).

**Conclusion**

Nous rapportons des preuves de perturbations de l'offre et de l'utilisation des soins de santé primaires pendant la pandémie de COVID-19 dans trois contextes distincts : Goma, RDC ; Kambia, Sierra Leone ; et Masaka, Ouganda. Nous avons constaté une diminution de 20 à 50 % des consultations pour certains services essentiels de soins de santé primaires. L'ampleur de la perturbation et les services touchés différaient selon les contextes. Le niveau de perturbation semble être en corrélation avec la force des mesures de confinement dans les différents contextes et avec l'attitude de la communauté vis-à-vis du risque posé par le COVID-19, en particulier dans les contextes ayant un historique de réponses aux épidémies d'Ebola. Des stratégies d'atténuation telles que des campagnes de communication sur la santé et des services de proximité se sont avérées importantes pour limiter l'impact des mesures de confinement sur les services de soins de santé primaires.

**Remerciements**

Nous remercions les équipes de collecte de données, le personnel des établissements de santé et les responsables des ministères de la santé des pays étudiés pour avoir facilité la collecte de ces données.

**Contributions des auteurs**

**Conception et réalisation du projet :** K Gallagher, D Watson-Jones, B Greenwood, B Leigh, H Kavunga, E Ruzagira.

**A collecté/généré les données du projet :** K Kasonia, D Tindanbil, J Kitonsa, F Zalwango, L Enria, A Mansaray, M James, Y Nije, D Tetsa Tata, B Lawal, A Drammeh, D Mukadi.

**A contribué aux données/outils analytiques :** Kathy Baisley

**A effectué l'analyse :** K Gallagher

**Projet initial :** K. Kasonia, D Tindanbil

**Révision et examen de toutes les ébauches :** K Kasonia, D Tindanbil, J Kitonsa, K Baisley, F Zalwango, L Enria, A Mansaray, M James, Y Nije, D Tetsa Tata, B Lawal, A Drammeh, B Lowe, D Mukadi, S Mounier-Jack, F Nakiyimba, P Obady, J Muhavi, B Greenwood, D Samai, B Leigh, D Watson-Jones, H Kavunga, E Ruzagira, K Gallagher.

**Financement**

Ce projet a été financé par une subvention de recherche UKRI (MRC), DHSC (NIHR) (GEC1017, MR/V029363/1, PI Katherine Gallagher).

**Conflit d'intérêts**

Tous les auteurs : aucun conflit d'intérêt signalé

**Légendes des figures**

**Figure 1. Le changement en pourcentage des niveaux d'activité en 2020 par rapport à 2019, pour chaque résultat, dans chaque période, à Goma^1^**

^1^ Le pourcentage de changement a été calculé comme suit : (1-RR_2020/2019_ )*100 ; les RR pour 2018 vs 2019 et 2019 vs 2020 sont inclus dans le tableau supplémentaire 1.

**Figure 2. Le changement en pourcentage des niveaux d'activité en 2020 par rapport à 2019, pour chaque effet, dans chaque période, à Kambia, Sierra Leone.^1^**

^1^ Le pourcentage de changement a été calculé comme suit : (1-RR_2020/2019_ )*100 ; les RR pour 2018 vs 2019 et 2019 vs 2020 sont inclus dans le tableau supplémentaire 2.

**Figure 3. Le changement en pourcentage des niveaux d'activité en 2020 par rapport à 2019, pour chaque résultat, dans chaque période, à Masaka, en Ouganda.^1^**

**Références**

1. Organisation mondiale de la santé. Enquête pulsée sur la continuité des services de santé essentiels pendant la pandémie de COVID-19. Rapport intérimaire. 27 août. Organisation mondiale de la santé ; 2020.

2.l' Organisation mondiale de la santé. Second tour de l'enquête nationale sur la continuité des services de santé essentiels pendant la pandémie de COVID-19. 23 avril 2021. 2021. Rapport n° : WHO/2019-nCoV/EHS_continuity/survey/2021.1.

3. Hale T, Angrist N, Goldszmidt R, Kira B, Petherick A, Phillips T, et al. A global panel database of pandemic policies (Oxford COVID-19 Government Response Tracker). Nature Human Behaviour. 2021;5(4):529-38.

4. Lim J, Broughan J, Crowley D, O'Kelly B, Fawsitt R, Burke MC, et al. COVID-19's impact on primary care and related mitigation strategies : A scoping review. Eur J Gen Pract. 2021;27(1):166-75.

5. Vaccaro C, Mahmoud F, Aboulatta L, Aloud B, Eltonsy S. The impact of COVID-19 first wave national lockdowns on perinatal outcomes : a rapid review and meta-analysis. BMC Pregnancy and Childbirth. 2021;21(1):676.

6. McDonald HI, Tessier E, White JM, Woodruff M, Knowles C, Bates C, et al. Early impact of the coronavirus disease (COVID-19) pandemic and physical distancing measures on routine childhood vaccinations in England, January to April 2020. Euro surveillance : bulletin européen sur les maladies transmissibles = European communicable disease bulletin. 2020;25(19).

7. Miller HE, Henkel A, Leonard SA, Miller SE, Tran L, Bianco K, et al. The impact of the COVID-19 pandemic on postpartum contraception planning. American journal of obstetrics & gynecology MFM. 2021;3(5):100412-.

8. Wanyana D, Wong R, Hakizimana D. Evaluation rapide de l'utilisation des services de santé maternelle et infantile pendant le COVID-19 au Rwanda. Action de santé publique. 2021;11(1):12-21.

9. Bell D, Hansen KS, Kiragga AN, Kambugu A, Kissa J, Mbonye AK. Prévision de l'impact de COVID-19 et de l'impact potentiel de la réponse de la santé publique sur la charge de morbidité en Ouganda. Am J Trop Med Hyg. 2020;103(3):1191-7.

10. COVID-19 ; PfE-BRt. Mise en œuvre effective des mesures sociales et de santé publique en Ouganda et en RD Congo : Analyse situationnelle. Deuxième collecte de données, 19 août 2020. Trouver l'équilibre : Santé publique et mesures sociales en Ouganda et en RD Congo : 3ème collecte de données. 2020.

Le gouvernement de Sierra Leone Ministère de la Santé et de l'Assainissement. Le Ministère de la Santé et de l'Assainissement, Sierra Leone <https://mohs.gov.sl/> [Dernier accès le 07 avril 2022] 2022 [

Le gouvernement de l'Ouganda. The Government of Uganda COVID-19 Response Information Hub https://covid19.gou.go.ug/timeline.html [dernier accès le 07 avril 2022] 2022 [

13. Harris PA, Taylor R, Minor BL, Elliott V, Fernandez M, O'Neal L, et al. The REDCap consortium : Building an international community of software platform partners. J Biomed Inform. 2019;95:103208.

14. Harris PA, Taylor R, Thielke R, Payne J, Gonzalez N, Conde JG. Research electronic data capture (REDCap)--une méthodologie basée sur les métadonnées et un processus de travail pour fournir un soutien informatique à la recherche translationnelle. J Biomed Inform. 2009;42(2):377-81.

15. Hannah Ritchie, Edouard Mathieu, Lucas Rodés-Guirao, Cameron Appel, Charlie Giattino, Esteban Ortiz-Ospina, et al. "Coronavirus Pandemic (COVID-19)". Publié en ligne sur OurWorldInData.org. Récupéré de : [" https://ourworldindata.org/coronavirus "](https://ourworldindata.org/coronavirus') [Ressource en ligne, dernier accès le 07 avril 22] 2020 [

16. Organisation mondiale de la santé. Rapport de situation : Epidémie d'Ebola 2018-2020 - Nord-Kivu-Ituri <https://www.who.int/emergencies/situations/Ebola-2019-drc-> [dernier accès le 07avril2022] 2021 [

Shet A, Carr K, Danovaro-Holliday MC, Sodha SV, Prosperi C, Wunderlich J, et al. Impact of the SARS-CoV-2 pandemic on routine immunisation services : evidence of disruption and recovery from 170 countries and territories. The Lancet Global Health. 2022;10(2):e186-e94.

18. Hategeka C, Carter SE, Chenge FM, Katanga EN, Lurton G, Mayaka SM-N, et al. Impact of the COVID-19 pandemic and response on the utilisation of health services in public facilities during the first wave in Kinshasa, the Democratic Republic of the Congo. BMJ Global Health. 2021;6(7):e005955.

19. Aranda Z, Binde T, Tashman K, Tadikonda A, Mawindo B, Maweu D, et al. Disruptions in maternal health service use during the COVID-19 pandemic in 2020 : experiences from 37 health facilities in low-income and middle-income countries. BMJ Global Health. 2022;7(1):e007247.

20. Namuganga JF, Briggs J, Roh ME, Okiring J, Kisambira Y, Sserwanga A, et al. Impact of COVID-19 on routine malaria indicators in rural Uganda : an interrupted time series analysis. Journal du paludisme. 2021;20(1):475.

21. Charles Onyango-Obbo. La migration urbaine-rurale ouvre les campagnes aux réfugiés du COVID-19. 09 mai 2020 [[](https://www.theeastafrican.co.ke/tea/oped/comment/urban-rural-migration-opens-up-countryside-to-covid-19-refugees--1441052)https://www.theeastafrican.co.ke/tea/oped/comment/urban-rural-migration-opens-up-countryside-to-covid-19-refugees--1441052]. 2020.

22. UNHABITAT. COVID-19 à travers le prisme des liens entre villes et campagnes - Principes directeurs et cadre d'action (URL-GP) [[](https://unhabitat.org/sites/default/files/2020/07/issue_brief_covid-19_through_the_lens_of_urban_rural_linkages_web_revised.pdf)https://unhabitat.org/sites/default/files/2020/07/issue_brief_covid-19_through_the_lens_of_urban_rural_linkages_web_revised.pdf]. 2020.
